# Long acting β_2 _agonists for stable chronic obstructive pulmonary disease with poor reversibility: a systematic review of randomised controlled trials

**DOI:** 10.1186/1471-2466-4-7

**Published:** 2004-08-31

**Authors:** Don Husereau, Vijay Shukla, Michel Boucher, Shaila Mensinkai, Robert Dales

**Affiliations:** 1 Canadian Coordinating Office for Health Technology Assessment (CCOHTA), 600-865 Carling Avenue, Ottawa ON K1S 5S8, Canada; 2 Health Research Institute, University of Ottawa, Ottawa ON K1H 8L6, Canada

## Abstract

**Background:**

The long acting β2-agonists, salmeterol and formoterol, have been recommended, by some, as first line treatment of stable chronic obstructive pulmonary disease (COPD). We reviewed evidence of efficacy and safety when compared with placebo or anticholinergic agents in patients with poorly reversible COPD.

**Methods:**

After searching MEDLINE, EMBASE, HealthSTAR, BIOSIS Previews, PASCAL, ToxFile, SciSearch, the Cochrane Library, and PubMed, as well as Web sites, selected journals, reference lists, and contacting drug manufacturers, two reviewers independently screened reports of randomised controlled trials of parallel or crossover design lasting four weeks or longer and including patients with a forced expiratory volume in one second (FEV1) ≤ 75% of predicted, a ratio of FEV1 to forced vital capacity (FVC) ≤ 88% of predicted, and < 15% improvement from baseline FEV1 after a dose of a β2 agonist. We included trials comparing salmeterol or formoterol with placebo or with ipratropium bromide and reporting one of these outcomes: lung function; exercise capacity; quality of life scores; dyspnea; exacerbations; rescue inhaler use; incidence of tachycardia, hypokalemia, or dry mouth. Two reviewers assessed the quality of included reports using the Jadad scale and allocation concealment, and abstracted data.

**Results:**

Twelve trials satisfied our inclusion criteria; eight were high quality (Jadad score >2) and four were low quality (≤ 2). The adequacy of allocation concealment was unclear in all of them. We did not perform a meta-analysis due to differences in trial design and how outcomes were reported.

Two trials comparing salmeterol with ipratropium did not detect differences; one trial comparing formoterol and ipratropium described greater improvement with formoterol in morning PEFR (15.3 versus 7.1 l/min, p = 0.040).

Of twelve trials comparing long acting β2 agonists with placebo, six reported no improvement in exercise capacity, eleven reported improvements in FEV1 lung function (one reported no improvement), six reported less rescue inhaler usage (one reported no difference) and five reported improved dyspnea scores (two reported no improvement). Differences in quality of life were detected in one salmeterol trial ; however, two salmeterol, and one formoterol trial reported no differences. Adverse effects of interest were not reported.

**Conclusion:**

In terms of clinical outcomes and safety, we could not find convincing evidence that salmeterol and formoterol have demonstrated advantages to ipratropium, a less expensive drug, for patients with stable COPD and poor reversibility. Compared to placebo, we found evidence of reduced rescue inhaler usage and improved spirometric outcomes without a significant impact on quality of life or exercise capacity.

## Background

Bronchodilators are the primary agents used to manage chronic obstructive pulmonary disease (COPD). They modestly improve forced expiratory volume in one second (FEV1) and reduce dynamic hyperinflation; breathlessness may lessen and exercise tolerance increase despite little improvement in spirometric measurements [1]. The bronchodilators currently available for COPD include β2 agonists (e.g., salbutamol and salmeterol), anticholinergics (e.g., ipratropium bromide) and methylxanthines (e.g., theophylline).

According to the Canadian guidelines for the treatment of stable COPD [2] first line treatment consists of ipratropium, two to four doses three to four times daily, plus a short acting β2 agonist administered on an "as needed" basis. If the patient uses substantial amounts of short acting β2 agonists, or if the symptoms are greater at night than in the early morning, a long acting β2 agonist (salmeterol or formoterol) is added twice daily. However, recently some have recommended the latter as first line agents for stable COPD [3], [4-7] potentially replacing the less expensive ipratropium [4], [5-8].

Several trials have demonstrated the usefulness of salmeterol and formoterol for the management of COPD[8], [9-11]. According to a 1998 meta-analysis [12], in patients with non-reversible COPD these agents produce small increases in FEV1; however, these changes alone may not correlate highly with symptom relief [13]. The authors of the meta-analysis suggested that these drugs be prescribed only for patients who find they provide definite clinical improvement: reduced breathlessness or better exercise capacity. All three trials in the meta-analysis [10,14-16]. compared long acting β2 agonists with placebo. Since then, other studies of these agents in COPD, including comparisons with anticholinergics [8,11] have appeared in the literature.

Canadian provincial drug plan managers have noted a substantial increase in the use of salmeterol and formoterol in recent years, an observation supported by data from International Medical Services Canada, which collects information on Canadian patterns of drug prescribing and estimates use: between 1997 and 2001, the use of salmeterol and formoterol for COPD increased 1,150% and 1,975%, respectively, whereas the use of ipratropium for COPD decreased by 37% [17].

In light of the new trials and the recent changes in prescribing practices, we undertook a systematic review to evaluate the efficacy and safety of long acting β2 agonists when compared with placebo or anticholinergic agents in patients with stable, poorly reversible, COPD.

## Methods

### Searching

We obtained published literature and conference abstracts for this document from two separate sources: (1) search results from the CCOHTA's published health technology review "Long-acting β2-agonists for maintenance therapy of stable chronic obstructive pulmonary disease: a systematic review"; and, (2) search results from CCOHTA's ongoing clinical review on long-acting β_2_-agonists for maintenance treatment of stable chronic obstructive pulmonary disease in mixed population. The first search was performed on MEDLINE^®^, EMBASE^®^, HealthSTAR, BIOSIS Previews^® ^in June 2001 using a sensitive search strategy. The second search performed in December 2002 had a more focused search strategy and included PASCAL, SciSearch and ToxFile databases in addition to MEDLINE^®^, EMBASE^® ^and BIOSIS Previews^® ^databases. As designed in the search strategy, this search captured all the studies included in CCOHTA's published review as well as some additional trials published since previous search date. Search details for both searches can be found in Appendix 2 [see Additional file 2]. Regular alerts have been established on these databases to capture new studies and are ongoing in 2004. Parallel searches were performed and updated in PubMed and the Cochrane Library. In addition, we periodically searched Web sites of clinical trial registries and health technology assessment (HTA) and related agencies. Google™ and other search engines were used to retrieve conference abstracts of major respiratory associations. We also hand searched selected journals and documents in the library of the Canadian Coordinating Office for Health Technology Assessment and the bibliographies of retrieved reports. As well, we contacted the Canadian offices of the manufacturers of salmeterol and formoterol for nonconfidential information on unpublished studies.

### Selection

Two reviewers (D R H and V K S) worked independently on these phases of the study. Disagreements were resolved by discussion and consensus; a neutral third party (M B) was consulted when necessary.

The reviewers evaluated the 504 unique citations by reviewing titles and abstracts, discarding those deemed irrelevant (e.g., case reports, review articles, and studies unrelated to the use of β2 agonists for maintenance treatment of stable COPD). They then selected all reports of randomised controlled trials (RCTs) comparing salmeterol or formoterol with placebo or an anticholinergic agent, with or without the additional use of short acting β2 agonists. No restrictions were placed on dosage, but the trials had to be of either parallel or crossover design, have lasted four weeks or longer, and have included patients that met each of the following criteria.

• Non-asthmatic.

• Stable COPD: no infections, exacerbations, or hospitalizations in the past month.

• FEV1 ≤ 75% of predicted.

• Ratio of FEV1 to forced vital capacity (FVC) ≤ 88% of predicted.

• After a dose of a short or long acting β2 agonist < 15% improvement in FEV1.

Since bronchodilators are much more efficacious in asthma than in COPD, including patients with asthma would have influenced the findings. It may be difficult to determine whether chronic airflow obstruction with relatively large responses to short acting β2 agonists represents COPD with reversibility or asthma with incomplete reversibility. A suggestive feature in the differential diagnosis of COPD is irreversible airflow limitation.[18] To better reflect this and to minimize the chance of including patients with asthma, we excluded those trials in which the average FEV1 response to a bronchodilator was greater than or equal to 15%.

In addition, the trials had to have investigated one of the following outcomes.

• Lung function, including FEV1 and peak expiratory flow rate (PEFR).

• Exercise capacity: six minute or shuttle walking test.

• Health related quality of life (QoL).

• Dyspnea, including symptom diary scores.

• Exacerbations of COPD.

• Rescue use of salbutamol, a short acting β2 agonist.

• Adverse effects, including tachycardia, hypokalemia, and dry mouth.

### Validity assessment

The reviewers independently scored the quality of the included trial reports using a five-point scale described by Jadad [19], which assigns zero to two points each for appropriateness of randomization and double blinding and zero to one point each for reporting on withdrawals and dropouts; low scores are associated with exaggerated estimates of benefit. Concealment of allocation to treatment was also categorized as adequate, inadequate, or unclear.

### Data abstraction

The reviewers independently recorded characteristics of the trials and patients, as well as details of the interventions and outcomes. When outcome data were available only graphically, each reviewer estimated values, and the means of the two estimates were reported.

### Quantitative data synthesis

When possible, we calculated mean differences with 95% confidence intervals (CIs) for continuous outcomes and odds ratios (ORs) with 95% CIs for binary outcomes for individual trial data using Statistics with Confidence software [20], We used intention-to-treat data when available and otherwise end point data for patients completing the trials. Qualitative data were recorded descriptively. We had intended to do a meta-analysis, pooling data on outcomes of interest. This approach is useful when the samples of individual studies are too small for detection of an effect and when results from several trials disagree in magnitude and direction of effect [21]. However, it is only appropriate when the trials are clinically homogeneous.

We found that even commonly measured outcomes, such as FEV1, could not be combined by meta-analysis because of differences in how they were reported. For example, in the six trials comparing salmeterol with placebo, FEV1 was reported as a mean change in percent predicted[16], a mean change overall[15], a mean difference between trial arms[10], no difference (without data)[22], baseline and overall FEV1 (after 24 hrs without medication)[8] and as an 0 to 12 hour area-under-the-curve (FEV1-AUC) function[23] We were not successful in obtaining more data from study authors. We also had concerns about the meta-analysis of data from trials of parallel and crossover design[24] and differences in spirometry protocols including allowable medications. Therefore, we decided on a best evidence synthesis approach [25] instead.

## Results

### Trial Flow

**Figure 1:**
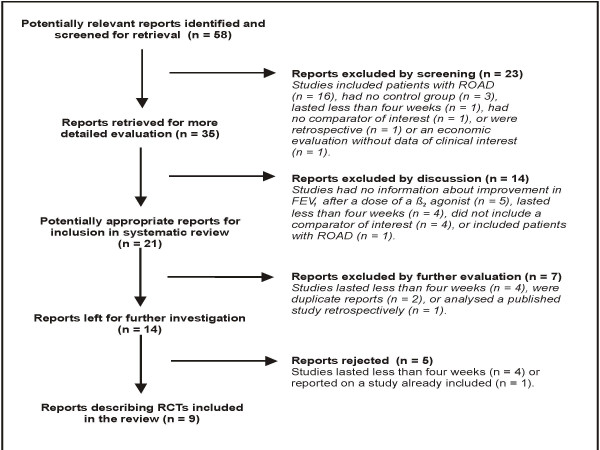
**Flow diagram of RCT screening and selection procedure. **Process through which reports were selected from those potentially relevant. RCT = randomised controlled trial; ROAD = reversible obstructive airways disease; FEV1 = forced expiratory volume in one second.

Both reviewers agreed to tentatively accept 35 of the 58 potentially relevant reports. After further evaluation one reviewer disagreed with including 14 of the 35, which resulted in a moderate level of agreement (Kappa = 0.58; 95% CI 0.39 to 0.78). Discussion revealed that this difference related primarily to confusion surrounding interpretation of one of the criteria for eligibility, and ultimately the other reviewer agreed to reject the disputed reports. The reviewers then independently selected the same nine reports [8,10,14-16,22,23,26,27] for final inclusion. Figure 1 illustrates the study selection process. The updated search strategy identified 24 additional potentially relevant reports. Of these, four reports were independently selected based on the inclusion criteria. There were no disagreements. Appendix 1 [see Additional file 1] presents all of the 69 reports excluded with reasons.

### Study characteristics

The 13 reports were of 12 trials, all funded by manufacturers of the drugs. One report [14] describes outcomes in a subset of patients fully described in another report [10]. One reports was a conference abstract [22]; the other twelve reports were journal articles. Duplicate reports were used as a source of supplementary information. Based on the reports, eight of the trials [8,15,16,23,26-29] were of high quality (score > 2) and four[10,22,30,31] of low quality (score ≤ 2). Concealment of the allocation sequence was unclear from all of the trial reports. The reviewers agreed completely about quality. Table 1 presents details of the trials and patients.

**Table 1:** Characteristics of included randomised, double blind, controlled trials of long acting β_2 _agonists in maintenance therapy for chronic obstructive pulmonary disease (COPD)

First author, year of publication, design	Trial quality	Patients meeting inclusion criteria	Interventions	Outcomes investigated	Notes
Ulrik, 1995[16] Crossover	3	66 current smokers with FEV_1 _of 1–2 L (< 60% of predicted) and FEV_1_/FVC < 60% of predicted. FEV_1_of <15% or 300 ml after salbutamol	Salmeterol (50 μg twice daily) or placebo for 4+4 weeks; no crossover washout.	FEV_1_, PEFR, daytime and night-time symptom scores, rescue use of salbutamol.	Two week run in. Methylxanthines, corticosteroids (short oral courses) allowed.
Newman, 1996[22] (abstract) Crossover	2	42 patients with mean FEV_1 _of 0.93 L (35% of predicted) and no response to oral steroids.	Salmeterol (100 μg twice daily) or placebo for 8+8 weeks.	FEV_1_, FVC, six minute walk test and Borg dyspnoea assessment,[26] daytime and night-time symptom scores, rescue use of salbutamol, proportion of days unable to perform normal activity, incidence of adverse events and COPD exacerbations.	Two week run in. Salbutamol rescue allowed.
Grove, 1996[15] Crossover	3	29 patients with FEV_1 _25%–75% of predicted and 5%–15% reversibility with 200 μg of salbutamol.	Salmeterol (50 μg twice daily) or placebo for 4+4 weeks; one1 week crossover washout.	FEV_1_, FVC, TLC, RV, 6 minute walk test and exertion on Borg scale, oxygen uptake.	At least one week run in. Inhaled corticosteroids, anticholinergics, oral theophylline allowed.
Boyd, 1997[10] Parallel	2	674 patients with FEV_1 _≤ 70% and FEV_1_/FVC ratio ≤ 60% of predicted and 5%–15% reversibility of FEV_1 _with 400 or 800 μg of salbutamol.	Salmeterol (50 or 100 μg twice daily) or placebo for 16 weeks.	FEV_1_, six minute walk test and Borg dyspnoea assessment, daytime and night-time symptom scores, rescue use of salbutamol.	Two week run in. Medications other than β_2 _agonists allowed.
Jones, 1997[14] Parallel	2	283 patients with FEV_1 _≤ 70% and FEV_1_/FVC ratio ≤ 60% of predicted; 5%–15% reversibility of FEV_1 _with 400 or 800 μg of salbutamol.	Salmeterol (50 or 100 μg twice daily) or placebo for 16 weeks.	HRQoL with SGRQ27 and SF-36[28].	Two week run in. Medications other than β_2 _agonists allowed.
Mahler, 1999[8] Parallel	3	145 patients with FEV_1 _≤ 65% and FEV_1_/FVC ratio ≤ 70% of predicted; ≤ 15% reversibility of FEV_1 _with short acting β_2_agonist; grade 1 baseline severity of breathlessness.	Salmeterol (42 μg twice daily) or ipratropium bromide (36 μg four times daily) or placebo for 12 weeks.	FEV_1 _AUC, six minute walk test, daytime and night-time symptom scores, dyspnoea on BDI and TDI,[29] supplemental use of salbutamol, HRQoL on CRDQ,[30] COPD exacerbations.	Run in six hours to three days. Prednisone (≤ 10 mg) or equivalent or inhaled corticosteroids allowed.
Rennard, 2001[23] Parallel	3	179 patients with FEV_1 _≤ 65% and FEV_1_/FVC ratio ≤ 70% of predicted; ≤ 12% reversibility of FEV_1 _with salbutamol; score ≥ 1 on MMRC five point dyspnoea scale.	Salmeterol (42 μg twice daily) or ipratropium (36 μg four times daily) or placebo for 12 weeks.	FEV_1 _and FVC AUC, dyspnoea on BDI and TDI, six minute walk test and Borg dyspnoea assessment, symptom scores, QoL on CRDQ, COPD exacerbations.	Corticosteroids, inhaled and oral (< 10 mg/d), allowed.
Rossi, 2002[27] Parallel	3	418 patients with FEV_1 _< 70% and FEV_1_/FVC ratio ≤ 88% of predicted; < 15% reversibility of FEV_1 _with short acting β_2_agonist; grade 1 baseline severity of breathlessness.	Formoterol (12 or 24 μg twice daily) or placebo or oral slow release theophylline for 12 months.	FEV_1 _AUC.	Inhaled corticosteroids and rescue use of salbutamol allowed.
Stahl, 2002[26] Parallel	3	183 patients with FEV_1 _< 60% and FEV_1_/FVC < 70% of predicted; < 12% reversibility of FEV_1 _after single dose of formoterol.	Formoterol (18 μg twice daily) or ipratropium (80 μg three times daily) or placebo for 12 weeks.	FEV_1_, FVC, PEFR, shuttle walking test, morning and evening symptom scores, HRQoL on SGRQ.	Inhaled corticosteroids at constant doses and rescue use of short acting β_2 _agonists allowed.
Gupta, 2002[29] Parallel	4	33 patients with FEV_1 _< 60 % predicted and FEV_1_/FVC ≤ 70%; reversibility <12 % improvement of FEV_1 _after 400 μg salbutamol	Salmeterol (50 μg twice daily) or placebo twice daily for 8 weeks	FEV_1_, FVC, six minute walk test, HRQoL on SF-36[28], dyspnoea on BDI, patient self-assessment, and rescure inhaler usage	Two week run in period. Patients required to take beclomethasone 400 μg twice daily and ipratropium 20 μg four times daily.
Mahler, 2002[30] Parallel	2	158 patients with FEV_1 _< 65 % predicted and FEV_1_/FVC ≤ 70%; reversibility <12 % improvement of FEV_1 _after 400 μg salbutamol	Salmeterol (50 μg twice daily) or placebo twice daily for 24 weeks	FEV_1_, morning PEF, dyspnoea on BDI and TDI; rescue salbutamol use; HRQoL on CRDQ [30]; symptoms on CBSQ	Randomization stratified by reversibility.
Calverly, 2003[28] Parallel	5	733 patients with FEV_1_25–70% predicted and FEV_1_/FVC ≤ 70%; reversibility <10 % of predicted FEV_1 _after salbutamol	Salmeterol (50 μg twice daily) or placebo twice daily for 52 weeks	FEV_1_, FVC, relief medication, symptom scores, night-time awakenings, exacerbation rates, HRQoL on SGRQ	Two week run in and two week follow up
Hanania, 2003[31] Parallel	2	163 patients with FEV_1 _< 65% predicted but > 700 ml (or if ≤ 700 ml > 40 % predicted) and FEV_1_/FVC < 65%; reversibility < 12 % of predicted FEV_1 _after salbutamol	Salmeterol (50 μg twice daily) or placebo twice daily for 24 weeks	FEV_1_, morning PEF, dyspnoea on BDI and TDI; rescue salbutamol use; HRQoL on CRDQ [30]; symptoms on CBSQ, exacerbation rates (all severities)	Randomization stratified by reversibility

AUC = area under the curve; BDI = baseline dyspnoea index;[29] CBSQ = chronic bronchitis symptom questionnaire; [42] CRDQ = chronic respiratory disease questionnaire;[30] FEV1 = forced expiratory volume in one second; FVC = forced vital capacity; HRQoL = health related quality of life; MMRC = Modified Medical Research Council; PEFR = peak expiratory flow rate; RV = residual volume; SF-36 = Medical Outcomes Study Short Form 36;[28] SGRQ = St. George's Respiratory Questionnaire;[27] TDI = transition dyspnoea index;[29] TLC = total lung capacity.

### Data synthesis

#### Comparative efficacy of long acting β2 agonists and anticholinergic agents

Three trials [8,23,26] that compared long acting β2 agonists and anticholinergic agents were identified. Two 12 week trials compared salmeterol, ipratropium, and placebo [8,23].; however, only one trial [8] reported data for FEV1 and transition dyspnea index (TDI) scores for the subset of patients that met our inclusion criteria, and the data were presented graphically. No significant differences (p > 0.05) between the salmeterol and ipratropium groups were observed in the change in FEV1 from baseline, in TDI scores, or in the rescue use of salbutamol [8].

In a 12 week trial [26] formoterol produced significantly greater improvement in morning PEFR from baseline to endpoint than ipratropium (15.3 versus 7.1 l/min, p = 0.040). However, the differences between the active treatment groups were not significant (p > 0.05) for percent predicted FEV1 (13% versus 7%, p > 0.05), percent predicted FVC (8% versus 8%, p > 0.05), improvement in breathlessness score (-0.21 versus -0.29, p > 0.05), or improvement in the St. George's Respiratory Questionnaire (SGRQ) total score (0.0 versus -0.5, p > 0.05). Data on adverse effects of interest, including tachycardia, hypokalemia, and dry mouth, were not available from the reports.

#### Comparative efficacy of long acting β2 agonists and placebo

Ten trials [8,10,14-16,22,23], [28-31] had salmeterol and placebo treatment arms; the other two [26,27]. had formoterol and placebo arms. Table 2 and the following text summarize outcome data only for the patients that met our inclusion criteria.

**Table 2:** Selected results

**First author**	**FEV**_1_	**Symptom scores (lower is better)**
***Salmeterol versus placebo***		

Ulrik[16]	No significant differences in reversibility of percent predicted FEV_1 _with treatment. Mean (SE): 2.7% (0.4) versus 3.4% (0.4).	Significant differences in median (range) symptom scores during treatment. Daytime (scale 0–5): 1.0 (0–3.4) versus 1.8 (0.1–4.0). Night-time (scale 0–4): 0.9 (0–3.4) versus 1.6 (0.1–4.0).
Newman[22]	No significant differences in measurements with treatment (data not reported).	Symptoms significantly reduced during salmeterol compared with placebo treatment. Scale and scores not reported.
Grove[15]	Significant differences one and six hours after single dose and six hours after four weeks of treatment. Mean change: 120 versus 10 ml after four weeks.	
Boyd[10]	Significant differences in improvement with treatment. Mean difference (95% CI): for salmeterol 50 μg versus placebo 97.80 (55.6 to 139.99) ml; for salmeterol 100 μg versus placebo 117.60 (67.88 to 167.32) ml.	Significant difference in distribution of median daytime and night-time symptom scores between active treatment and placebo groups (CI 0.0 to 0.0 in all cases) but not between active treatment groups. Daytime (scale 0–5): baseline, 2 in each group; from week 5, 1 in active treatment groups and 2 in placebo group. Night-time (scale 0–4): baseline, 1 in placebo and salmeterol 50 μg groups and 0 in salmeterol 100 μg group; from week 1, 0 in salmeterol 50 μg group and no change in other groups.
Jones[14]	(Presented QoL results for subset of patients described in Boyd[10].)	
Gupta[29]	A mean increase in predose FEV_1 _of 170 ml (distibution not reported) for salmeterol vs. a mean decrease of 20 ml (distribution not reported) for placebo after 8 weeks.	Both salmeterol and placebo produced significant improvemnts in BDI scores, however the magnitude of increase was greater vs. placebo (3 vs. 1); 100% patients treated with salmeterol reported decreased cough and dyspnea vs. 69% (11/16) of placebo recipients
Mahler 2002 [30]	A mean increase of 80 ml (95%CI 35 to 125) for salmeterol vs. mean decrease of -8 ml (95%CI: -53 to 37) for placebo. Two-hour post-dose FEV_1 _mean increase of 175 ml (95%CI: 116 to 234) vs. mean increase of 28 ml (95%CI: -17 to 73)	Mean increase of 0.5 (SE 0.4) in TDI for salmeterol recipients and 0.4 (SE 0.3) for placebo recipients. Not clinically or statistically significant.
Calverly [28]	A mean increase in predose FEV_1_of 25 ml vs. a mean decrease of -38 ml (P < 0.05) in salmeterol and placebo recipients. Smaller difference for two-hour post-dose FEV_1 _(data not reported).	Mean scores for cough (scale 0–3); breathlessness (scale 0 to 4); sputum production (scale 0 to 3); sputum colour (scale 0 to 4): salmeterol: cough 1.36 (SE0.03); breathlessness 1.59 (0.03); sputum production 1.30 (0.03) and colour 1.35 (0.03) vs. placebo: cough 1.44 (0.03); breathlessness 1.66 (0.03), sputum production 1.34 (0.03) and colour 1.36 (0.03).
Hanania[31]	A mean increase of 26 ml (95%CI: -27 to 79) for salmeterol vs. mean increase of 19 ml (95%CI: -26 to 64) for placebo. Two-hour post-dose FEV_1 _mean increase of 119 ml (95%CI: 70 to 168) vs. mean increase of 71 ml (95%CI: 24 to 118)	The magnitude of TDI responses was less in non-reversible vs. reversible patients. (Data are not reported)
**Salmeterol versus ipratropium versus placebo**		
Mahler[8]	Significant differences between active treatment and placebo groups but not between active treatment groups. Peak improvements with treatment: 155, 165, and 24 ml, respectively.	No significant differences in change of mean daytime symptom score with treatment. No significant differences in TDI except between ipratropium and placebo groups at week 8. After 12 weeks, mean TDI 0.35, 0.98, and 0.48, respectively.
Rennard[23]	Significant differences between active treatment and placebo groups but not between active treatment groups. FEV_1_AUC 0–12 hour responses significantly greater with salmeterol and ipratropium than with placebo (data not reported).	
**Formoterol versus placebo**		
Rossi[27]	Significant differences in estimated difference in FEV_1_AUC 0–12 hour responses: between formoterol 12 μg and placebo groups, 145 ml; between formoterol 24 μg and placebo groups, 141 ml. (Individual values for treatment groups not available.)	
**Formoterol versus ipratropium versus placebo**		
Stahl[26]	Significant differences in improvement in percent predicted FEV_1_between active treatment and placebo groups but not between active treatment groups: 13%, 7%, and 6%, respectively.	Significant differences between active treatment and placebo groups in change from baseline in breathlessness (scored 0 to 4 morning and evening). Means: -0.21, -0.29, and 0.0, respectively.

CI = confidence interval; SE = standard error.

##### Lung function

###### FEV1

As table 2 shows, the changes in FEV1 from baseline to endpoint differed significantly (p < 0.05) between the salmeterol and placebo groups in eight of ten trials and between the formoterol and placebo groups in two trials.

###### FVC

Five trials[15,23,26,28,31] reported on this outcome. In one 4+4 week (4 weeks then crossover then 4 additional weeks) trial [15] the increase in FVC was significantly greater with salmeterol than with placebo six hours after a single dose (200 versus 30 ml, 95%CI for difference: 40 to 290) but not after four weeks of treatment (150 versus 130 ml, 95%CI for difference: -180 to 220). In one 12 week study [23] the change in FVC was significantly greater (p < 0.001) for salmeterol (and ipratropium) than for placebo on day 1, there was no loss of response during treatment, and after four weeks the morning predose values were significantly greater in the patients treated with either active drug (data not reported). In the other 12 week trial [26] the percent predicted FVC was significantly increased by the end of formoterol treatment, compared with placebo treatment, by 8% versus -0.4% (p = 0.02). In one 52 week trial).)[28], the change in mean FVC measured 12 hours after treatment was 86 ml greater (p = 0.004) in salmeterol recipients. The difference in mean change in FVC at 52 weeks was 200 ml between groups. In an 8 week trial[29], the mean increase in FVC was 280 ml in the salmeterol group compared to a fall of 8 ml in the placebo group (p < 0.05).

###### PEFR

Three trials[16,26,28].).) reported on this outcome. In one four week trial [16] salmeterol treatment compared with placebo treatment produced a mean treatment difference in morning values of 12 l/min (238 compared with 226 l/min, 95%CI for difference: 6 to 17; p < 0.001); a statistical difference for evening values was not detected [242 (95%CI: 222 TO 262) and 237 (95% CI: 217 to 257) l/min for salmeterol and placebo, p > 0.1]. The diurnal variation was significantly lower during salmeterol treatment, at 3 (95%CI: -0.9 to 6.9) versus 11 (95%CI: 7.1 to 14.9) l/min; however, the mean treatment difference was only 7 (95%CI: 3 to 11) l/min. In the other trial, lasting 12 weeks [26], the change in morning PEFR was significantly greater by the end of formoterol (or ipratropium) treatment compared with placebo treatment: 15.3 versus -0.9 l/min (p < 0.001). In one 52 week trial).)[28], the change in mean PEF values differed significantly (p < 0.0001) for salmeterol treatment, at 257 l/min (95%CI: 253 to 261) versus placebo, at 242 l/min (95%CI: 238 to 246).

##### Exercise capacity

Results (but not always data) for six minute walk tests were reported from six trials [8,10,15,22,26,29]. None of the trials found statistically significant differences between salmeterol and placebo therapy, although one 12 week trial[8] found that at week 10 the patients receiving ipratropium walked a mean of 14 (95%CI: 0.3 to 27.7) yards farther in six minutes than those receiving placebo; there were no differences in prewalk or postwalk breathlessness between the treatment groups. The only other trial reporting data [15] found a median (interquartile range) distance in six minutes of 450 (371–491) m for placebo recipients and 425 (392–473) m for salmeterol recipients; the difference was not reported to be significant, but the patients receiving salmeterol (50 μg twice daily) perceived significantly less exertion by the end of treatment, as measured on the Borg scale [median (interquartile range) 0.5 (0–1) for salmeterol versus 1 (0–2) for placebo, p = 0.004] [32]. A 16 week trial [10] found a significant (p < 0.05) reduction in postwalk breathlessness (three or more points on the 10 point Borg scale) after eight and 16 weeks of 50 μg but not 100 μg of salmeterol twice daily, compared with placebo (OR 0.62 [95% CI 0.42 to 0.91]). Similarly, an 8+8 (8 weeks then crossover then 8 additional weeks) week trial [22] did not detect a significant (p > 0.05) difference in postwalk breathlessness between patients receiving 100 μg of salmeterol and those receiving placebo.

One study comparing formoterol, ipratropium, and placebo [26] reported mean changes in walking distance from baseline to endpoint, measured with the shuttle walking test, of 19.2, 17.5 and 5.1 m, respectively; the differences were not significant (p > 0.05).

##### Dyspnea

In several trials [10,16,22,26,28-31] the patients assessed symptom severity every day, generally using ordinal scales. One 12 week trial comparing salmeterol, ipratropium, and placebo [8] measured the severity of dyspnea at baseline with a multidimensional baseline dyspnea index (BDI) and changes in severity every two weeks with a TDI [33]. As table 2 shows, some differences during treatment with an active drug as compared with placebo were significant and others were not.

##### Rescue use of a short acting β2 agonist

In five of six trials salmeterol treatment was associated with less salbutamol use than was placebo treatment [10,16,22,28,29] In one trial (4+4 weeks) [16] the median numbers (range) of daytime rescue doses were 1.7 (0–6.1) and 2.6 (0–7.9), respectively, and the median numbers of night-time doses 0 (0–4.2) and 0.3 (0–5.0). In a 52 week trial.[28], the median number of rescue inhalations per day was 2 for both salmeterol and placebo recipients, but these groups were statistically different (p = 0.028). Another trial[29] reported the mean number of doses of rescue salbutamol was significantly lower during treatment in salmeterol recipients (0.59, 95%CI: 0.30 to 0.88) versus placebo recipients (1.75, 95%CI: 1.33 to 2.17).

In one 12 week trial comparing salmeterol, ipratropium, and placebo [8] no significant difference was observed in additional bronchodilator use between the placebo and active drug groups.

##### Quality of life

HRQoL was evaluated in four trials[14,26,28,29] In a salmeterol study [14] a subset of a larger patient group was asked to complete the disease-specific SGRQ [34] and the Medical Outcomes Study Short Form 36 (SF-36) [35] at baseline and after 16 weeks of treatment. The SGRQ has three components: distress due to respiratory symptoms, effects of disturbances on mobility and physical activity, and psychosocial impact of the disease; negative changes represent improvement. Data from 283 patients (95 in the placebo group and 94 in each salmeterol group) were analysed; data for others were excluded because of noncompletion of one or both questionnaires at 16 weeks or inability to meet quality control criteria or both. Salmeterol 50 μg (but not 100 μg) twice daily was associated with significantly greater improvement in mean (standard deviation) SGRQ scores from baseline to endpoint than was placebo: -6.8 (13.2) versus -1.4 (11.7) for total score and -8.0 (17.6) versus 0.0 (15.7) for impact score. No significant differences between placebo and either dose of salmeterol were observed in any of the domains of the SF-36 except for "role-emotional": these scores were significantly worse for recipients of salmeterol 100 μg than for recipients of placebo.

In the 52 week study).)[28], health status was assessed with the SGRQ. The adjusted mean score was not statistically different in salmeterol recipients, at 45.2 (95%CI: 44.4 to 46.0) versus placebo recipients, at 46.3 (95%CI: 45.3 to 47.2). In an 8 week study[29] the magnitude of improvement for salmeterol versus placebo recipients rated on an SF-36 scale was significantly greater for the dimensions of "general health" (p = 0.008), "health change" (p = 0.026); physical functioning" (p = 0.008) and "vitality energy and fatigue" (p = 0.008)

In the trial comparing formoterol, ipratropium, and placebo [26] HRQoL was also evaluated with the disease specific SGRQ. Of the 183 patients, 144 completed the assessment; reasons for not doing so were not reported. The changes from baseline to endpoint in total score were negligible in all three groups, at 0.0, -0.5, and 1.5, respectively.

##### COPD exacerbations

Three trials[10,22,28] reported on this outcome; only one trial).)[28] defined "COPD exacerbation" as episodes that required antibiotics or corticosteroids but not hospital admission; these occurred at a mean rate of 0.54 exacerbations/patient/year in salmeterol recipients and 0.76 exacerbations/patient/year in placebo recipients (p = 0.0003). In one 16 week trial [10] the numbers (and proportions) of patients having exacerbations among those receiving salmeterol 50 or 100 μg twice daily or placebo were 75 (33%), 91 (42%), and 98 (43%), respectively. In an 8+8 week trial [22] there were fewer exacerbations during treatment with salmeterol 100 μg twice daily than during treatment with placebo (p = 0.065); data were not presented.

##### Adverse Effects

Data on adverse effects of interest were not available from the reports.

## Discussion

We identified thirteen reports of twelve randomized controlled trials describing the effect of administering the long acting β2-agonists, salmeterol and formoterol, to patients with poorly reversible COPD.

It is not clear from the reports whether the twelve selected trials had sufficient power to detect significant differences between treatment and control groups in the various subjective and objective outcome measures. Since data were not pooled for meta-analysis, we were not able to conduct a sensitivity analysis based on the quality of trial reporting. Accordingly, we cannot comment on the possible influence of quality on the effect size of the outcome measures. Clinical heterogeneity among the trials limited assessment of the overall effect of the interventions. Since we did not perform a meta-analysis, statistical heterogeneity was not an issue.

We selected reports that met our inclusion criteria, regardless of publication status, language and trial quality using a systematic research methodology; this approach has been shown to minimize potential selection and publication bias and lead to more reliable conclusions[36] We made every effort to conduct our review and report its results with the highest rigour.

A potential limitation of our research is that we did not seek trials comparing long acting β2-agonists marketed outside of Canada (e.g., bambuterol) or those trials comparing long acting β2-agonists to agents other than ipratropium and placebo (e.g., short-acting β2-agonists, methylxanthines). Similarly, we excluded those trials in which the FEV1 response to a bronchodilator was not reported or greater than 15%. Thus, our results may not be generalisable to the greater population of patients who can be currently defined as having COPD[18]. We plan to include a greater number of comparators and a broader population in an upcoming analysis[37]

Our results are similar to those of an earlier review [12] that identified three placebo controlled trials included in our review, but there are two important differences. In the earlier review FEV1 endpoint data from the placebo and salmeterol groups in two crossover trials [15,16] were pooled; the weighted mean differences were not significant. We preferred to analyse net improvement in FEV1 (the difference from baseline to endpoint), as we felt that it more accurately reflected the impact of maintenance therapy. In addition, no trials comparing long acting β2 agonists and ipratropium were available at the time of the earlier review.

Another review has recently been published.[38] However, these authors restricted their search to MEDLINE and failed to identify a clinical trial comparing formoterol with ipratropium.[26] As a consequence, the evidence describing the use of formoterol versus ipratropium is limited to a single trial.[39] In contrast, we opted to exclude this trial after identifying two trials because roughly 40% of patients exhibited partial reversibility of FEV1 (15% to 80%) to an inhaled dose of 200 mcg salbutamol at baseline. We are in agreement with the authors' summary of the evidence surrounding salmeterol versus ipratropium.

We believe our findings are in accord with current guidelines, such as the GOLD guidelines, that suggest bronchodilators should be prescribed according to individual patient responses. However, policymakers with limited health service resources need to be aware of an identifiable sub-population of patients with poorly reversible COPD for which long acting β2-agonists may result in reduced efficiency (cost-effectiveness).

Our research also suggests clinical investigators of COPD trials should stratify trial participants into groups for which outcomes may consistently differ. Of the trials identified, four[23,30,31,40] used this approach. Outcome information from patients with poor reversibility was also analyzed in an abstract[41] of an excluded trial but not in the published report.[39] We were unable to ascertain sufficient details surrounding this analysis to add it to our findings.

## Conclusions

In terms of clinical outcomes and safety, we could not find convincing evidence that salmeterol and formoterol have demonstrated advantages to ipratropium, a less expensive drug, for patients with stable COPD and poor reversibility. Compared to placebo, we found evidence of reduced rescue inhaler usage and improved spirometric outcomes without a significant impact on quality of life or exercise capacity.

## Competing interests

Donald Husereau, Vijay Shukla, Michel Boucher, and Shaila Mensinkai have no competing interests to declare. CCOHTA is an independent, nonprofit health research agency funded by the federal, provincial, and territorial governments of Canada. Robert Dales sits on the advisory committees for GlaxoSmithKline (makers of the long acting β2 agonist, salmeterol) and Boeheringer Ingelheim (makers of the anticholinergic agent, ipratropium bromide).

## Authors' contributions

DH edited and prepared the final manuscript for publication. VS led development of the research protocol, supervised the literature review, and summarized results. DH and VS were responsible for reviewing articles, judging their relevance, assessing their quality, and extracting data. MB assisted in developing the research protocol and in conflict resolution during study selection. RD assisted in developing the research protocol and provided clinical expertise. SM designed and conducted the electronic searches and provided expertise in the area of information science. All authors either wrote sections or critically reviewed drafts of this article.

## Pre-publication history

The pre-publication history for this paper can be accessed here:



## Supplementary Material

Additional File 1 Appendix 1. A list of reports considered in this review but excluded. Click here for file

Additional File 2 Appendix 2. Search strategies including databases, time horizons and subject headings/keywords used to locate trials. Click here for file
