# Supplementary material for: Long acting β2 agonists for stable chronic obstructive pulmonary disease with poor reversibility: a systematic review of randomised controlled trials
Source: BMC Pulm Med. 2004 Aug 31;4:7. doi: 10.1186/1471-2466-4-7 (PMC517721; doi:10.1186/1471-2466-4-7)
Supplement: Additional File 1 — Appendix 1. A list of reports considered in this review but excluded. [file 1471-2466-4-7-S1.pdf]

Appendix 1 Excluded reports

Studies related to reversible obstructive airways disease

1. Beeh KM, Wiewrodt R, Salem AE, *et al*. Wirksamkeit und verträglichkeit von salmeterol in der langzeitthe-rapie bei patienten mit obstruktiver atemwegserkrankung. SAL-MULTI-Studiengruppe. *Pneumologie* 2000;**54**:225-31.

2. Çelik G, Kayacan O, Beder S, *et al*. Formoterol and salmeterol in partially reversible chronic obstructive pulmonary disease: a crossover, placebo-controlled comparison of onset and duration of action. *Respiration* 1999;**66**:434-9.

3. Thomson NC, Angus R, Quebe-Fehling E, *et al*. Efficacy and tolerability of formoterol in elderly patients with reversible obstructive airways disease. *Respir Med* 1998;**92**:562-7.

4. van Noord JA, de Munck DR, Bantje TA, *et al*. Long-term treatment of chronic obstructive pulmonary disease with salmeterol and the additive effect of ipratropium. *Eur Respir J* 2000;**15**:878-85.

5. Cazzola M, Di Perna F, Centanni S, *et al*. Acute effect of pretreatment with single conventional dose of salmeterol on dose–response curve to oxitropium bromide in chronic obstructive pulmonary disease. *Thorax* 1999;**54**:1083-6.

6. Patakas D, Andreadis D, Mavrofridis E, *et al*. Comparison of the effects of salmeterol and ipratropium bromide on exercise performance and breathlessness in patients with stable chronic obstructive pulmonary disease. *Respir Med* 1998;**92**:1116-21.

7. Cazzola M, Di Perna F, Noschese P, *et al*. Effects of formoterol, salmeterol or oxitropium bromide on airway responses to salbutamol in COPD. *Eur Respir J* 1998;**11**:1337-41.

8. Ramirez-Venegas A, Ward J, Lentine T, *et al*. Salmeterol reduces dyspnea and improves lung function in patients with COPD. *Chest* 1997;**112**:336-40.

9. Schultze-Werninghaus G. Multicenter 1-year trial on formoterol, a new long-acting β_2-agonist, in chronic obstructive airway disease. *Lung* 1990;**168 Suppl**:83-9.

10. D'Urzo AD, Chapman KR, Cartier A, *et al*. Effectiveness and safety of salmeterol in nonspecialist practice settings. *Chest* 2001;**119**:714-9.

11. Vervloet D, Ekström T, Pela R, *et al*. A 6-month comparison between formoterol and salmeterol in patients with reversible obstructive airways disease. *Respir Med* 1998;**92**:836-42.

12. Cazzola M, Santangelo G, Piccolo A, *et al*. Effect of salmeterol and formoterol in patients with chronic obstructive pulmonary disease. *Pulm Pharmacol* 1994;**7**:103-7.

13. Schmitz E, Hürter T, Ochs J-G, *et al*. Steigerung der körperlichen Leistungsfähigkeit unter Wirkung von Salmeterol. *Pneumologie* 1994;**48**:12-5.

14. Germouty J, Aubert J, Clavier J, *et al*. Tolérance à long terme du formotérol chez des bronchopathies chroniques obstructifs. *Allerg Immunol (Paris)* 1992;**24**:342-7.

15. Weiner P, Magadle R, Berar-Yanay N, *et al*. The cumulative effect of long-acting bronchodilators, exercise, and inspiratory muscle training on the perception of dyspnea in patients with advanced COPD. *Chest* 2000;**118**:672-8.

16. Cazzola M, Di Perna F, Califano C, *et al*. Formoterol Turbuhaler (F) vs salmeterol Diskus (S) in patients with partially reversible stable COPD [abstract]. *Am J Respir Crit Care Med* 1999;**159 (3 Suppl)**:A798.

17. Condemi JJ. Comparison of the efficacy of formoterol and salmeterol in patients with reversible obstructive airway disease: a multicenter, randomized, open-label trial. *Clin Ther* 2001;**23**:1529-41.

Studies not satisfying or stipulating a criterion of < 15% improvement in forced expiratory volume in one second after a dose of a β_2 agonist

1. Konermann M, Suchantke R, Mogck J, *et al*. Formoterol monotherapy compared with combined ipratropium bromide plus fenoterol in the treatment of chronic obstructive pulmonary disease. *Clin Drug Invest* 2001;**21**:235-42.

2. Chapman K, Kuipers AF, Goldstein R, *et al.* Addition of salmeterol 50 mcg *bid* to anticholinergic treatment in COPD [abstract]. *Am J Respir Crit Care Med* 1999;**159 (3 Pt 2 Suppl)**:A523.

3. Melani AS, Pirrelli M, Di Gregorio A. Effects of inhaled salmeterol and orally dose-titrated theophylline on exercise capacity of stable COPD patients [abstract]. *Eur Respir J Suppl* 1996;**9 (Suppl 23)**:391S.

4. Ulrik CS. Airway responsiveness in COPD: effect of salmeterol? [abstract]. *Eur Respir J Suppl* 1998;**12 (Suppl 28)**:1S.

5. Dahl R, Greefhorst LA, Nowak D, *et al*. Inhaled formoterol dry powder versus ipratropium bromide in chronic obstructive pulmonary disease. *Am J Respir Crit Care Med* 2001;**164**:778-84.

6. Watkins M, Wire P, Yates J, Fischer T, Chang C, Horstman D. Sustained FEV1 increases in COPD patients induced by salmeterol 50mcg twice daily via the diskus inhaler [abstract]. Am J Respir Crit Care Med 2002;**165(8 Suppl)**:A228.

7. Dahl R, Greefhorst AP, Thomson MH, Till D. Formoterol (foradil®) improves lung function and quality of life (QOL) parameters in patients with reversible or poorly reversible COPD [abstract]. Am J Respir Crit Care Med 2001;**163(5 Suppl)**:A280.

8. Donohue JF, van Noord JA, Bateman ED, Langley SJ, Lee A, Witek TJ, et al. A 6-month, placebo-controlled study comparing lung function and health status changes in COPD patients treated with tiotropium or salmeterol. Chest 2002;**122(1)**:47-55.

9. Brusasco V, Hodder R, Miravitlles M, Korducki L, Towse L, Kesten S. Health outcomes following treatment for six months with once daily tiotropium compared with twice daily salmeterol in patients with COPD. Thorax 2003;**58(5)**:399-404.

10. Eliraz A, Bengtsson T, Bogdan M, Coenen PDM, Johansson G, Osmanilev D, et al. **Formoterol (Oxis) Turbuhaler is effective and safe as maintenance or maintenance plus reliever therapy in patients with COPD [poster].** In: ATS 2003 - 99th International Conference; 2003 May 16-2003 May 21. Seattle (WA): American Thoracic Society; 2003.

11. Bogdan M, Eliraz A, Mckinnon C, Nihlen U, Radeczky E, Soliman S, et al. **Formoterol Turbuhaler is an effective maintenance and maintenance plus reliever therapy in patients with chronic obstructive pulmonary disease (COPD) irrespective of the level of lung function impairment and reversibility [poster].** In: ATS 2003 - 99th International Conference; 2003 May 16-2003 May 21. Seattle (WA): American Thoracic Society; 2003.

12. Donohue JF, Rea HH, Menjoge SS, Kesten S. **Alterations in bronchodilator effectiveness over six months with tiotropium and salmeterol [poster].** In: ATS 2003 - 99th International Conference; 2003 May 16-2003 May 21. Seattle: American Thoracic Society; 2003.

13. Friedman M, Morera G, Menjoge S, Kesten S. **Reduced COPD exacerbations with tiotropium [poster].** In: ATS 2003 - 99th International Conference; 2003 May 16-2003 May 21. Seattle: American Thoracic Society; 2003.

14. Szafranski W, Cukier A, Ramirez A, Menga G, Sansores R, Nahabedian S, et al. Efficacy and safety of budesonide/formoterol in the management of chronic obstructive pulmonary disease. Eur Respir J 2003;**21(1)**:74-81.

Studies not satisfying or stipulating a criterion of< 75% FEV_1 predicted

1. Dal Negro RW, Pomari C, Tognella S, Micheletto C. Salmeterol & fluticasone 50 microg/250 microg bid in combination provides a better long-term control than salmeterol 50 microg bid alone and placebo in COPD patients already treated with theophylline. Pulm Pharmacol Ther 2003;**16(4)**:241-6.

Studies lasting less than four weeks

1. Sichletidis L, Kottakis J, Marcou S, *et al*. Bronchodilatory responses to formoterol, ipratropium, and their combination in patients with stable COPD. *Int J Clin Pract* 1999;**53**:185-8.

2. Kaushik ML, Kashyap S, Bansal SK, *et al*. Effectiveness of salmeterol in stable COPD. *Indian J Chest Dis Allied Sci* 1999;**41**:207-12.

3. Maesen BL, Westermann CJ, Duurkens VA, *et al*. Effects of formoterol in apparently poorly reversible chronic obstructive pulmonary disease. *Eur Respir J* 1999;**13**:1103-8.

4. Khoukaz G, Gross NJ. Effects of salmeterol on arterial blood gases in patients with stable chronic obstructive pulmonary disease. Comparison with albuterol and ipratropium. *Am J Respir Crit Care Med* 1999;**160**:1028-30.

5. Cazzola M, Imperatore F, Salzillo A, *et al*. Cardiac effects of formoterol and salmeterol in patients suffering from COPD with preexisting cardiac arrhythmias and hypoxemia. *Chest* 1998;**114**:411-5.

6. Cazzola M, Matera MG, Di Perna F, *et al*. A comparison of bronchodilating effects of salmeterol and oxitropium bromide in stable chronic obstructive pulmonary disease. *Respir Med* 1998;**92**:354-7.

7. Vollmer M, Schmidt EW, Ulmer WT. Wirkdauer und Wirkstärke von Salmeterol, Fenoterol und Salbutamol bei schweren Formen obstruktiver Atemwegserkrankungen. *Pneumologie* 1995;**49**:528-34.

8. Langley S, Woodcock A, Jones SW, *et al*. A placebo controlled comparison of the effect of single doses of salmeterol and the combination bronchodilator Combivent on lung function over 12 hours in patients with chronic obstructive pulmonary disease (COPD) [abstract]. *Thorax* 1999;**54 (Suppl 3)**:A65.

9. Cazzola M, di Marco F, Boveri B, *et al*. **Bronchodilating effect of a combination with salmeterol and zafirlukast in patients with chronic obstruction of the airways: a pilot study [abstract]**. In: *European Respiratory Society Annual Congress abstracts on disk;* Oct 9-13 1999; Madrid, Spain. European Respiratory Society;1999.

10. Ayers ML, Mejia RA, Ward J, *et al*. Comparison of salmeterol (42µg) and ipratropium bromide (72µg) on dynamic hyperinflation and dyspnea during exercise in patients with COPD [abstract]. *Am J Respir Crit Care Med* 2000;**161 (3 Suppl):**A749.

11. Refini RM, Sestini P, Alfano S, *et al*. **Effect of inhaled salmeterol or combined treatment with salbutamol and ipratropium bromide on the exercise tolerance in patients with severe chronic obstructive pulmonary disease [abstract]**. In: *European Respiratory Society Annual Congress abstracts on disk;* Oct 9-13 1999; Madrid, Spain. European Respiratory Society; 1999.

12. Tutluoglu B, Gürbüz N, Sahin S, *et al*. Effects of short term usage of formoterol on six-minute walking test in COPD patients [abstract]. *Eur Respir J Suppl* 1997;**10 (Suppl 25):**65S.

13. Ayers ML, Mejia R, Ward J, *et al*. Effectiveness of salmeterol versus ipratropium bromide on exertional dyspnea in COPD. *Eur Respir J* 2001;**17**:1132-7.

14. Corsico A, Fulgoni P, Beccaria M, Zoia MC, Barisione G, Pellegrino R, et al. Effects of exercise and beta 2-agonists on lung function in chronic obstructive pulmonary disease. J Appl Physiol 2002;**93(6)**:2053-8.

Drug not compared with comparator of interest

1. D'Urzo AD, De Salvo MC, Ramirez-Rivera A, *et al*. In patients with COPD, treatment with a combination of formoterol and ipratropium is more effective than a combination of salbutamol and ipratropium: a 3-week, randomized, double-blind, within-patient, multicenter study. *Chest* 2001;**119**:1347-56.

2. ZuWallack RL, Mahler DA, Reilly D, *et al*. Salmeterol plus theophylline combination therapy in the treatment of COPD. *Chest* 2001;**119**:1661-70.

3. Melani AS, Di Gregorio A. Terapia di lunga durata con salmeterolo per via inalatoria vs. teofillina orale a lento rilascio: effetti sulla saturazione ossiemoglobinica durante il test del cammino in pazienti con BPCO severo. *Lotta Contro Tuberc Malat Polm Sociali* 1994;**64**:331-6.

4. Di Gregorio A, Melani AS, Saporiti G. Salmeterolo versus teofillina nel trattamento della bronchite cronica. Analisi statistica. *Lotta Contro Tuberc Malat Polm Sociali* 1994;**64**:305-8.

5. Nagano H, Nishimoto Y, Egashira Y, *et al*. Double-blind comparative study of formoterol for chronic obstructive diseases, mainly for chronic bronchitis and pulmonary emphysema in multiple institutions. *Igaku Ayumi* 1984;**129**:578-94.

6. van Noord JA, Aumann J, Janssens E, Mueller A, Cornelissen PJG. **Comparison of once daily tiotropium, twice daily formoterol and the free combination, once daily, in patients with COPD [poster].** In: ATS 2003 - 99th International Conference; 2003 May 16-2003 May 21. Seattle (WA): American Thoracic Society; 2003.

Studies without control group

1. Del Torre L, Melica EV, Del Torre M. Effectiveness of salmeterol in patients with emphysema. *Curr Ther Res Clin Exp* 1992;**52**:888-98.

2. Ohta Y, Goto I. Clinical experience with formoterol (BD 40A) in chronic bronchitis. *Igaku Yakugaku* 1982;**7**:1205-9.

3. Shima K, Takenaka S. [The clinical evaluation of formoterol (BD 40A) in bronchial asthma with long term administration and in chronic obstructive pulmonary disease with short term administration]. *Yakuri Chiryo* 1983;**11**:3935-42.

Duplicate studies

1. Ulrik CS. Effekten af salmeterol i behandlingen af rygere med kronisk obstruktiv lungesygdom. *Ugeskr Laeger* 1996;**158**:3604-7.

2. Greefhorst A, Dahl R, Nowak D, *et al*. Effect of inhaled formoterol and ipratropium bromide on quality of life, "bad days" and exacerbations in patients with COPD [abstract]. *Am J Respir Crit Care Med* 2000;**161 (3 Suppl)**:A490.

3. Wilson K, Karia N, Sondhi S. Salmeterol xinafoate improves lung function and provides more symptom-free nights in chronic obstructive pulmonary disease (COPD) at marginal costs [abstract]. *Am J Respir Crit Care Med* 2000;**161 (3 Suppl)**:A489.

4. Wadbo M, Löfdahl CG, Larsson K, Skoogh BE, Tornling G, Arwerström E, et al. Effects of formoterol and ipratropium bromide in COPD: a 3-month placebo-controlled study. Eur Respir J 2002;**20(5)**:1138-46.

5. Boyd G, Crawford C. Salmeterol (SALM) for treatment of patients with chronic obstructive pulmonary disease (COPD) [abstract]. Eur Respir J 1995;**8 Suppl 19**:167S.

6. Dal Negro R, Micheletto C, Trevisan F, Tognella S, Pomari C. **Salmeterol & fluticasone 50µg/250µg bid vs salmeterol 50µg bid and vs placebo in the long-term treatment of COPD [abstract].** Am J Respir Crit Care Med 2002;165(8 Suppl):A228. Available: http://www.abstracts-on-line.com/abstracts/ATS/search/results.asp?Num=0%2E4788889 (accessed 2003 Apr 24).

7. Calverley PMA, Pauwels RA, Vestbo J, Jones PW, Pride NB, Gulsvik A, et al. **Salmeterol/fluticasone propionate combination for one year provides greater clinical benefit than its individual components [abstract].** Am J Respir Crit Care Med 2002;165(8 Suppl):A226. Available: http://www.abstracts-on-line.com/abstracts/ATS/search/results.asp?Num=0%2E7688364 (accessed 2003 Apr 24).

8. Kristufek P, Levine B, Till D, Byrne A. **Inhaled formoterol (foradil®) improves lung function in patients with both reversible and poorly reversible COPD [abstract].** Am J Respir Crit Care Med 2001;163(5 Suppl):A280.

9. Grove A, Lipworth BJ, Ramage L, Smith R, Ingram CG, Reid P, et al. **Effects of regular salmeterol on lung function and exercise capacity in patients with partially reversible COPD [abstract].** Eur Respir J Suppl 1995;8 Suppl 19:94S.

10. Jones PW, Edin HM, Anderson J. **Salmeterol/fluticasone propionate combination improves health status in COPD patients [abstract].** Am J Respir Crit Care Med 2002;165(8 Suppl):A111. Available: http://www.abstracts-on-line.com/abstracts/ATS/search/results.asp?Num=0%2E9193186 (accessed 2003 Apr 24).

11. Ulrik CS. **The efficacy of inhaled salmeterol in the management of smokers with moderate to severe chronic obstructive pulmonary disease (COPD) [abstract].** Eur Respir J Suppl 1995;8 Suppl 19:392S.

Retrospective studies

1. Donohue J, Emmett A, Rickard K, *et al*. Salmeterol is effective bronchodilator therapy for all stages of COPD [abstract]. *Am J Respir Crit Care Med* 1999;**159 (3 Pt 2 Suppl)**:A817.

2. Mahler D, ZuWallack R, Rickard K, *et al*. Effects of salmeterol and ipratropium on dyspnea as measured by the 6 minute walk and baseline dyspnea index/transitional dyspnea index (BDI/TDI) [abstract]. *Am J Respir Crit Care Med* 1997;**155 (4 Abstract Suppl)**:A278.

Economic evaluation without data of clinical interest

1. Wilson K, Karia N, Sondhi S. Salmeterol xinafoate improves lung function and provides more symptom-free nights in chronic obstructive pulmonary disease (COPD) at marginal costs [abstract]. *Am J Respir Crit Care Med* 2000;**161 (3 Suppl)**:A489.
