# Supplementary material for: Long acting β2 agonists for stable chronic obstructive pulmonary disease with poor reversibility: a systematic review of randomised controlled trials
Source: BMC Pulm Med. 2004 Aug 31;4:7. doi: 10.1186/1471-2466-4-7 (PMC517721; doi:10.1186/1471-2466-4-7)
Supplement: Additional File 2 — Appendix 2. Search strategies including databases, time horizons and subject headings/keywords used to locate trials. [file 1471-2466-4-7-S2.pdf]

Search Strategy#1
Long-acting ß$_2$-agonists for maintenance therapy of stable chronic obstructive pulmonary disease: a systematic review

DATABASES	LIMITS	SUBJECT HEADINGS/KEYWORDS
DIALOG OneSearch®<br><br>EMBASE® (1974 –2001)/Jun Week 3<br><br>MEDLINE® 1966 –2001/ June Week 4<br><br>HealthSTAR® 1975 – 2000/Dec<br><br>BIOSIS Previews® 1969 – 2001/Jun Week 3	Human	Chronic Obstructive Lung Disease/de OR Obstructive Airway Disease/de OR Chronic Obstructive Pulmonary Disease/de OR Lung Emphysema/de OR Bronchitis/de OR Chronic Bronchitis/de OR Asthma!/de OR Emphysema/de OR Lung Diseases, Obstructive!/de OR Bronchitis!/de OR Pulmonary Emphysema!/de OR Airway Obstruction!/de OR Emphysema! /de OR Mediastinal Emphysema/de OR Subcutaneous Emphysema/de OR Obstructive Lung Disease/de OR Chronic Obstructive Pulmonary Disease/de OR Asthma/de OR Emphysema/de OR Bronchitis/de OR COPD/ti,ab OR COAD/ti,ab OR chronic(w)obstructive(w)lung/ti,ab OR chronic(w)obstructive(w)airway?/ti,ab OR chronic(w)obstructive(w)pulmonary /ti,ab OR bronchit?/ti,ab OR bullous(w)disease?/ti,ab OR emphysema/ti,ab OR lung(w)emphysema/ti,ab or pulmonary(w)emphysema/ti,ab or asthma/ti,ab OR *Pulmonary Disease, Chronic Obstructive!/de (added in alerts and updates as it was introduced in 2002)*<br><br>**AND**<br>long(w)acting /ti,ab AND *(combine long-acting with drug class terms)*<br>[(Bronchodilator Agents!/de OR Adrenergic Beta-Agonists!/de OR Receptors, Adrenergic, Beta-2!/de OR Adrenergic(1N)beta/ti,ab AND (receptor? or agonist?) /ti,ab OR beta(w)2(w)adrenoceptor(w)agonist? /ti,ab OR bronchodilator?/ti,ab)] OR<br><br>Foradil /ti,ab OR foradile /ti,ab OR oxis /ti,ab OR eolus/ti,ab OR atock/ti,ab OR neblik/ti,ab OR serevent/ti,ab OR aeromax/ti,ab OR arial/ti,ab OR salmetedur/ti,ab OR beglan/ti,ab OR betamican/ti,ab OR inaspir/ti,ab OR salmeter/ti,ab OR serobid/ti,ab OR servent/ti,ab OR formoterol/ti,ab OR formoterol(w)fumarate /ti,ab OR salmeterol(w)xinafoate/ti,ab OR salmeterol/ti,ab OR eformoterol/ti,ab OR eformoterol(w)fumarate/ti,ab OR bambuterol/ti,ab OR bambec/ti,ab OR

DATABASES	LIMITS	KEYWORDS/SUBJECT HEADINGS
		RN=73573-87-2 OR RN=43229-80-7 OR RN=94749-08-3<br><br>**AND**<br><br>Controlled Study/de OR Meta Analysis/de OR Randomized Controlled Trial/de OR Single-blind Method/de OR Double-blind Method/de OR Meta-Analysis/de OR Random Allocation/de OR DT=Meta-Analysis OR DT=Randomized Controlled Trial OR DT=Controlled Clinical Trial OR clinical trial/de OR randomized trial/de OR prospective study/de OR randomized controlled trial/de OR multicenter study/de OR randomized clinical trial/de OR random?/ti,ab OR [(single OR double OR triple OR treble)()(blind? OR dumm? or mask?)]/ti,ab OR placebo?/ti,ab or meta()analy?/ti,ab OR metaanaly?/ti,ab OR (quantitative?(w)(review? OR overview))/ti,ab OR (methodologic?(w)(review? OR overview?))/ti,ab OR control?(w)stud?/ti,ab OR control?(w)trial?/ti,ab OR RCT?/ti,ab OR control?(w)clinical(w)stud?/ti,ab OR control?(w)clinical(w)trial?/ti,ab<br><br>**AND**<br><br>Human? OR people?OR person?<br><br>*Search performed 25/06/2001. Weekly DIALOG® alerts set up until 2002 to capture new references. Total = 504 unique hits*<br><br>*MEDLINE® = 323 hits*<br>*HealthSTAR® = 54 hits*<br>*EMBASE® = 122 hits*<br>*BIOSIS Previews® = 5 hits*
The Cochrane Library, 2001, 2002 & PubMed	Human	Same MeSH headings and keywords as DIALOG MEDLINE® search. Drug class terms, trade names, numbers and the clinical filter excluded in the Cochrane Library. Appropriate syntax used to search Cochrane Library and PubMed.

Search Strategy#2
Long-acting ß$_2$-agonists for maintenance treatment of stable chronic obstructive pulmonary disease: a systematic review of clinical studies

**DATABASES**	**DATES/LIMITS**	**SUBJECT HEADINGS/KEYWORDS**
DIALOG OneSearch®<br><br>EMBASE®<br><br>PASCAL<br><br>MEDLINE®<br><br>BIOSIS Previews®<br><br>ToxFile<br><br>SciSearch®	Human	Chronic Obstructive Lung Disease/de OR Chronic Obstructive Pulmonary Disease/de OR Lung Emphysema!/de OR Chronic Bronchitis/de OR Lung Diseases, Obstructive/de OR Pulmonary Disease, Chronic Obstructive!/de OR Bronchitis!/de OR Pulmonary Emphysema!/de OR Emphysema! /de OR Obstructive Lung Disease/de OR COPD/ti,ab OR COAD/ti,ab OR bronchit?/ti,ab OR lung(4N)emphysema/ti,ab OR pulmonary(4N)emphysema OR chronic(4N)obstructive<br><br>*(Copd terms combined with Asthma terms)*<br><br>**AND**<br>Asthma!/de OR asthma?/ti,ab OR Wheezing/deOR Respiratory Sounds/de OR Bronchial Hyperactivity/de OR wheez?/ti,ab<br>*(Results from COPD terms "OR"ed with Results from COPD+ASTHMA terms)*<br><br>**AND**<br>(*Combine drug class terms with long-acting)*<br>[(Bronchodilating Agent/de OR Beta Adrenergic Receptor Stimulating Agent/de OR Bronchodilator-Drug/de OR Beta-Adrenergic Receptor/de OR Beta-Adrenergic Agonists-Drug/de OR Beta-Adrenergic Agonists/de OR Bronchodilator Agents!/de OR Adrenergic Beta-Agonists!/de OR Receptors, Adrenergic, Beta-2!/de)] AND long(1N)acting /ti,ab OR<br><br>Salmeterol/de OR Formoterol/de OR Formoterol Fumarate/de OR Bambuterol/de OR Salmeterol Xinafoate/de OR Salmeterol/id OR Formoterol/id OR Bambuterol/id OR<br><br>RN=73573-87-2 OR RN=43229-80-7 OR RN=94749-08-3

DATABASES	LIMITS	SUBJECT HEADINGS/KEYWORDS
	Human	OR serobid/ti,ab OR servent/ti,ab OR serevent/ti,ab OR formoterol/ti,ab OR formoterol(w)fumarate /ti,ab OR optrol/ti,ab OR salmeterol(w)xinafoate/ti,ab OR salmeterol/ti,ab OR eformoterol/ti,ab OR eformoterol(w)fumarate/ti,ab OR bambuterol/ti,ab OR bambec/ti,ab OR oxcol/ti,ab OR GR()33343x/ti,ab OR long(1N)acting()beta(1N)2<br><br>**AND**<br><br>Controlled Study!/de OR Meta Analysis/de OR Randomized Controlled Trials/de OR Single-blind Method/de OR Epidemiologic Research Design!/de OR Double-blind Method/de OR Meta-Analysis/de OR Random Allocation/de OR Clinical Trials!/de OR Clinical Trial!/de OR Prospective Studies/de OR Prospective Study/de OR Randomized Controlled Trial/de OR Multicenter Study/de OR Randomized Clinical Trial/de OR Comparative Study/de OR Randomized Trial/de OR Phase 1 Clinical Trial/de OR Phase 2 Clinical Trial/de OR Phase 3 Clinical Trial/de OR Phase 4 Clinical Trial/de OR Follow-up Studies/de OR DT=Meta-Analysis OR DT=Randomized Controlled Trial OR DT=Controlled Clinical Trial OR random?/ti,ab OR placebo? OR meta()analy? OR metaanaly? OR RCT? OR<br><br>((control? OR clinical) (3N) (stud? OR trial?)) OR<br><br>((single OR double OR triple OR treble) () (blind? OR dumm? OR mask?)) OR<br><br>((quantitative? OR methodologic? OR systematic?) () (review? OR overview? OR synthes?s OR integration)) OR<br>((crossover OR cross(1N)over OR prospective OR cohort OR parallel) () (stud? OR trial? OR design))<br><br>**AND**

DATABASES	LIMITS	SUBJECT HEADINGS/KEYWORDS
		(Limit retrieval to human studies)<br><br>Human? OR people? OR person? ?<br><br>*Performed 03 Dec 2002 Regular alerts set up on MEDLINE, EMBASE, BIOSIS Previews, Pharmaceutical News Indix and Adis Clinical Trials Insigh. Alerts are still ongoing in 2004.*<br><br>*Total 297 unique records*<br>*Medline 110*<br>*Biosis 9*<br>*Embase 171*<br>*Pascal 6*<br>*Toxfile 1*
The Cochrane Library, Issue 4, 2002, all issues 2003.	Human	MeSH and keywords to mirror DIALOG® MEDLINE search excluding numbers which are ignored by the software. Clinical trial filter also excluded.<br><br>*The Cochrane Database of Systematic Reviews = 4 reviews; Protocols = 5; The Cochrane Controlled Trials Register = 109 references; INAHTA and other health care technology agencies = 2 abstracts*
PubMed<br>2002, 2003, 2004	Human	MeSH and keywords to mirror DIALOG® MEDLINE search
Websites of health technology assessment and related agencies; clinical trial registries; other databases		AHRQ; National Research Register; University of York NHS Centre for Reviews and Dissemination – CRD databases; LILACS, etc; Websites of professional associations such as, European Respiratory Society, Canadian Respiratory Society etc.
